# Vasomotion and Neurovascular Coupling in the Visual Thalamus *In Vivo*

**DOI:** 10.1371/journal.pone.0028746

**Published:** 2011-12-09

**Authors:** Casto Rivadulla, Carmen de Labra, Kenneth L. Grieve, Javier Cudeiro

**Affiliations:** 1 Laboratory of Neuroscience and Motor Control (Neurocom), Department of Medicine-INEF-Galicia, University of Coruña, Coruña, Spain; 2 Institute of Biomedical Research of Coruña (INIBIC), Coruña, Spain; 3 Faculty of Life Sciences, University of Manchester, Manchester, United Kingdom; University of Alberta, Canada

## Abstract

Spontaneous contraction and relaxation of arteries (and in some instances venules) has been termed vasomotion and has been observed in an extensive variety of tissues and species. However, its functions and underlying mechanisms are still under discussion. We demonstrate that *in vivo* spectrophotometry, measured simultaneously with extracellular recordings at the same locations in the visual thalamus of the cat, reveals vasomotion, measured as an oscillation (0.14hz) in the recorded oxyhemoglobin (OxyHb) signal, which appears spontaneously in the microcirculation and can last for periods of hours. During some non-oscillatory periods, maintained sensory stimulation evokes vasomotion lasting ∼30s, resembling an adaptive vascular phenomenon. This oscillation in the oxyhaemoblobin signal is sensitive to pharmacological manipulation: it is inducible by chloralose anaesthesia and it can be temporarily blocked by systemic administration of adrenaline or acetylcholine (ACh). During these oscillatory periods, neurovascular coupling (i.e. the relationship between local neural activity and the rate of blood supply to that location) appears significantly altered. This raises important questions with regard to the interpretation of results from studies currently dependent upon a linear relationship between neural activity and blood flow, such as neuroimaging.

## Introduction

First described in veins of the bat wing [1] and since observed in numerous species (including human) in different states of consciousness, in preparations including both *in vivo* and *in vitro,* and using different experimental approaches, vasomotion has been defined as any spontaneous rhythmic contraction and relaxation of arteries and in some instances venules. In the brain, vasomotion has been described in awake [2] and anaesthetized animals [3]–[5], and in *in vitro* preparations [6]. Nevertheless, vasomotion remains a phenomenon which is both imperfectly understood, and even relatively unknown (for a review see references [7]–[9]. While it is clear that blood vessels within the peripheral and central circulation are physiologically different, the phenomenon of vasomotion has been reported in both. Vasomotion requires coordinated activity of vascular smooth muscle cells, since oscillations in the membrane potential of muscle cells appear to precede oscillations in the vessel [10]–[11]. It is likely that this oscillatory activity in the membrane potential originates from an oscillatory release of calcium from intracellular stores, which may then activate calcium dependent ion channels on the cell membrane [12]–[14]. The coordination between muscle cells is then achieved through gap junctions [11]–[12], [15].

A major suggestion for the role of vasomotion is that it regulates blood flow at branch points [16], limiting the amount of blood to areas of the vascular network, but maintaining flow at low perfusion. It resembles other low frequency oscillations, such as the low frequency Blood Oxygen Level Dependent (BOLD) fluctuations observed in fMRI during rest state (see [17] for a review) which has been used as an indicator of functionally connected networks in the brain [18] and as a clinical marker for several brain pathologies [17].

Neurovascular coupling was originally defined as the regional variation in blood supply in response to local variation in neural activation [19] and it is generally assumed that the relationship is causal and linear [20]–[21]. Here we have used continuous spectrophotometric oxyhaemoglobin recordings to show that thalamic oxygen supply can oscillate independently of the level of spontaneous or driven neuronal activity measured by simultaneous extracellular recordings. This oscillatory state can last for hours and during these periods the vascular response to sensory stimulus is altered while neuronal responses remain robust, resulting in a noticeable change in thalamic neurovascular coupling. These results have consequences for the analysis of imaging results, many of which are based on a close coupling between neuronal and hemodynamic responses, and should also be kept in mind when trying to understand the interactions between the different elements of the nervous system, such as neurons, glia and associated blood vessels, collectively known as a neurovascular unit (for a recent review see reference [22]).

## Results

Spontaneous vasomotion of the thalamic microcirculation was observed using spectrophotometry in 10 cats. Experiments lasted for up to 2 days and vasomotion was equally prevalent in the first or second day of the experiment.

### Oscillatory pattern at rest

**Figure 1:**
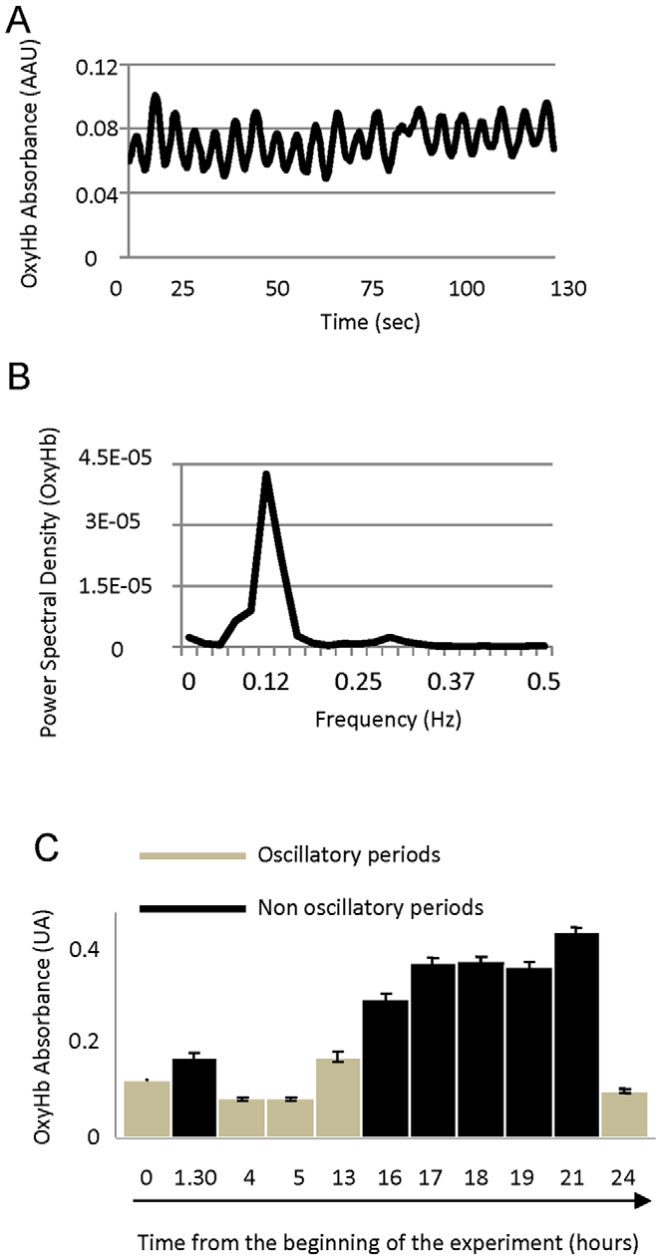
Vasomotion at the thalamic vasculature. A) Raw data showing the oscillatory OxyHb absorbance recorded during a period without visual stimulation. B) Power spectrum analysis of the signal shown in A. C) Average of the OxyHb signal during oscillatory and non-oscillatory episodes recorded in the same animal over ∼24 consecutive hours. In this case the oscillatory activity started spontaneously about 6 hours after the beginning of the experiment and lasted (with one short non-oscillating period) for 15 hours.

In the absence of sensory stimulation, the spectroscopic signal for the OxyHb showed two clearly different states: oscillatory and non-oscillatory. The oscillatory pattern is consistent with a continuous slow oscillation in the diameter of the vessels. Oscillatory frequency was on average 8.4 times per minute (0.14 Hz, range from 0.1 to 0.22). Figure 1 shows a characteristic recording where an oscillatory pattern is clearly visible (A) and the power spectral analysis of the raw signal showing the dominant frequency (B), in this example 0.12 Hz. We could not predict, based on the other physiological measured parameters (e.g. ECG, EEG) if the OxyHb signal would oscillate or not. Spontaneous oscillatory and non oscillatory periods alternated during the course of the experiments. Duration of the oscillatory periods varied from minutes up to several hours, and these were generally accompanied by a decrease in basal OxyHb levels (figure 1C). In the experiment represented in figure 1C the oscillation was present at the beginning of the experiment and lasted, with a single short interruption, for 15 hours. In this experiment, observed basal values of OxyHb were on average 67% lower during the oscillatory than during the non-oscillating period. Pooled results from 8 animals showed an average decrease in OxyHb signal of 34% during oscillations.

Oscillatory vasomotion did not seem to be restricted spatially, since different consecutive electrode tracks at different stereotaxic locations in the dorsal lateral geniculate nucleus (dLGN) found oscillatory activity. Penetrations were separated up to 1 mm, in the same hemisphere. Spontaneous neuronal activity did not show oscillations in the frequency range observed in the OxyHb signal. We used autocorrelation analysis on spontaneous neuronal activity recorded simultaneously with the OxyHb signal and coherence analysis between neuronal and OxyHb recordings. No relationship between the signals was found (data not shown).

### Oscillation and visual stimulation

**Figure 2:**
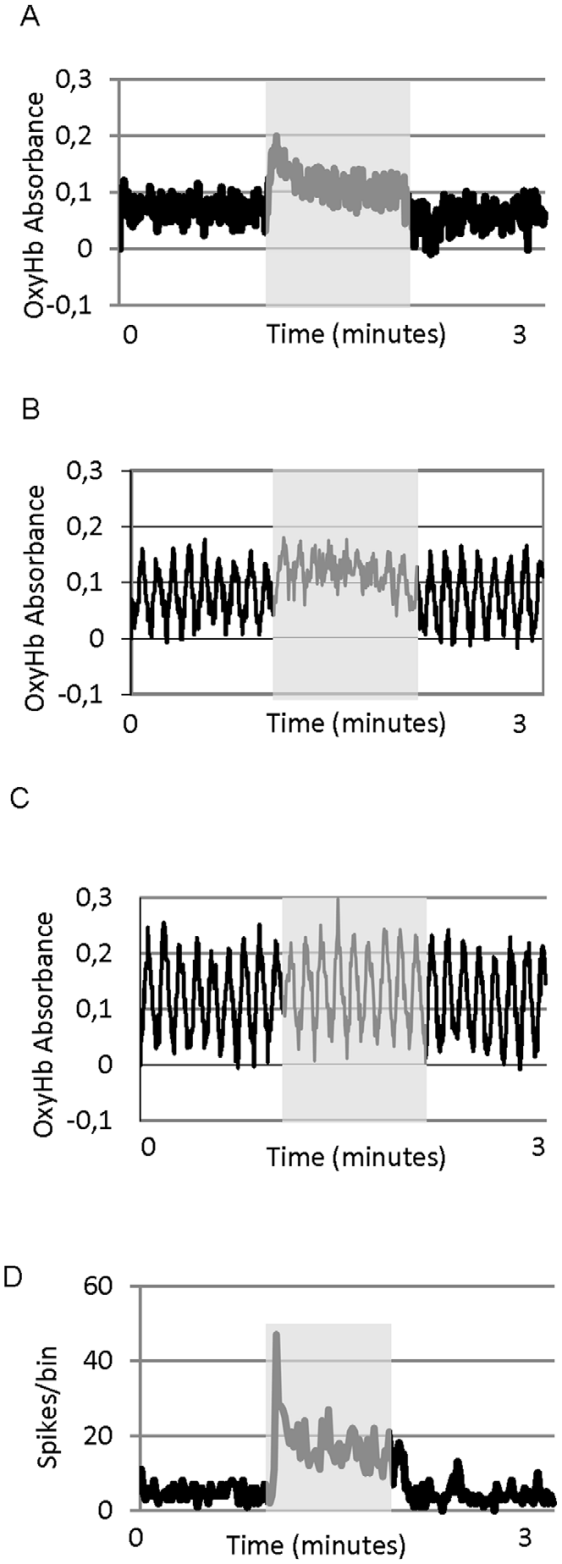
Vasomotion interferes with neurovascular coupling. A) Raw data showing the change in OxyHb during visual stimulation (shaded area) in a non-oscillating recording (A), and during spontaneous oscillatory periods (B and C). D) Multiunit neuronal activity recorded simultaneously to the OxyHb signal shown in C.

During non-oscillatory periods, visual stimulation evoked a noticeable, rapid and stable increase in the OxyHb absorbance: 29.8% ±3 SEM on average (n = 60; p<0.05). During oscillatory periods the increase in OxyHb absorbance evoked by sensory stimulation ranged from normal to none. This is illustrated in figure 2 with 3 recordings from the same animal during non oscillatory signal (A), weak (B) and strong (C) oscillation. Figure 2D shows multiunit neuronal activity recorded simultaneously with the OxyHb signal shown in fig. 2C. Here, there are clear visual responses (increased firing during stimulus presentation, shaded area) even in the absence of an increase in OxyHb signal.

**Figure 3:**
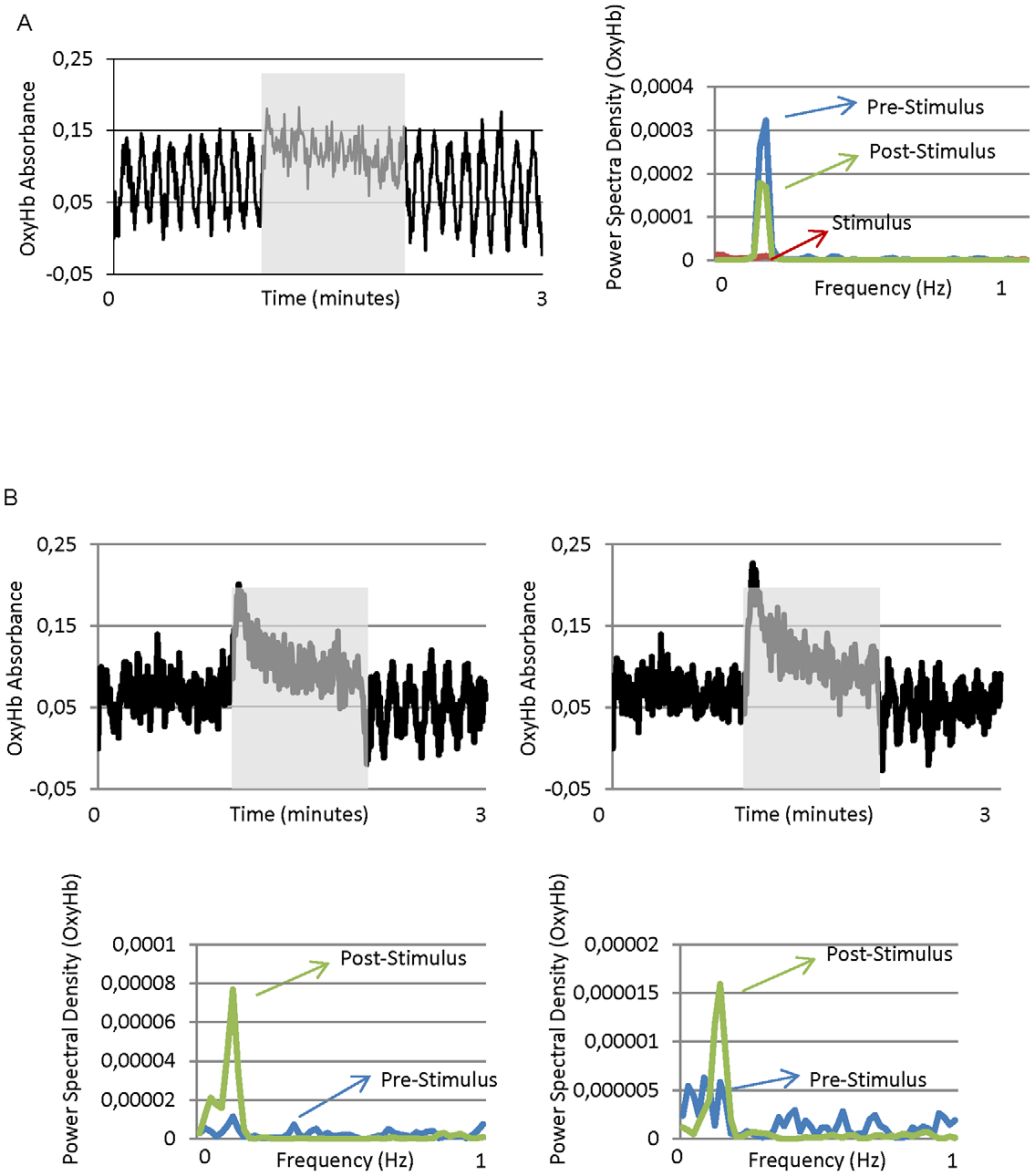
Sensory stimulation affects vasomotion. A) Raw OxyHb data (left) and power spectral analysis of the OxyHb signal (right). For power spectral density analysis (PSD) the signal has been separated into pre-stimulus, stimulus (shaded area) and post-stimulus epochs. B) Two consecutive recordings (5 minutes separation) of OxyHb showing the appearance of an oscillatory pattern after the stimulus stopped. PSD analysis for both recordings: periods before and after stimulus presentation are shown at the bottom of the figure.

Sensory stimulation had two principal effects on oscillation in the OxyHb signal (Fig. 3). Firstly, during periods of spontaneous oscillation, stimulus presentation reduced or even abolished the oscillation, which reappeared immediately after stimulus disappeared (Fig. 3A, also Fig. 2B). Paradoxically, during some periods of non-oscillation in which the stimulus presentation evoked an increase in OxyHb, the end of stimulation evoked a clear oscillatory activity that lasted for about 40 sec. Figure 3B shows two consecutive recordings showing this effect.

### Induction of oscillatory signals and neurovascular uncoupling

**Figure 4:**
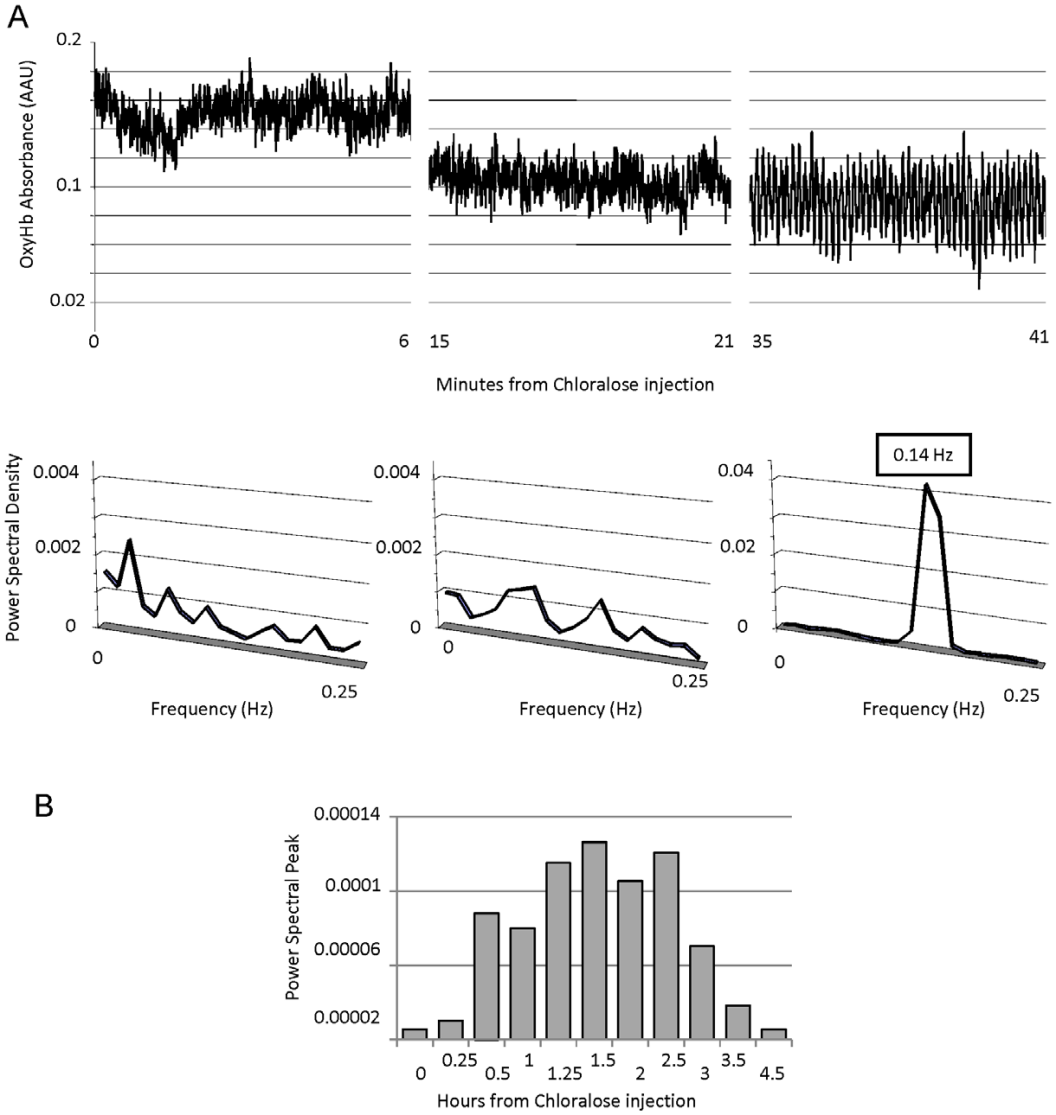
Chloralose induced vasomotion. A) Raw recordings of OxyHb signal (top) and power spectral analysis (bottom) at intervals after the chloralose injection. B) Evolution of oscillatory strength after chloralose injection. Power spectral peak is represented versus time.

Slow-wave vasomotion is frequently unpredictable and difficult to reproduce, but there is evidence suggesting that anesthesia can affect the presence of oscillations (see discussion). Increasing the level of isoflurane up to 3.5% in 2 different animals had clear effects on other recorded physiological parameters, (including a decrease of evoked neuronal and OxyHb signals) but did not induce oscillations in the spectrophotometric signal (Figure S1). Chloralose administration (60 mg/kg, i.v., see methods) induced profound vasomotion in recordings in 2 animals, which persisted for the entire period of anesthetic activity (Fig. 4). Figure 4A shows the temporal evolution of the OxyHb signal in the absence of sensory stimulation, from time 0 (chloralose injection) to 35 minutes later. The left column shows the initial 6 minutes recording immediately after injecting chloralose and ceasing isoflurane administration and the power spectrum analysis of the signal (lower). The centre column shows the signal 15 min after the injection - there is an evident decrease in the basal level of OxyHb but no oscillatory activity. The right column shows the recording 35 minutes after the injection - the oscillatory pattern is now clearly visible, and is reflected in the power spectrum analysis, showing a peak at 0.14 Hz (notice the change in scale in Y axis). Oscillation reached maximum strength about 1 hour after the injection (figure 4B) and remained the predominant peak in the power spectrum for the next 4 hours, with no change in frequency.

**Figure 5:**
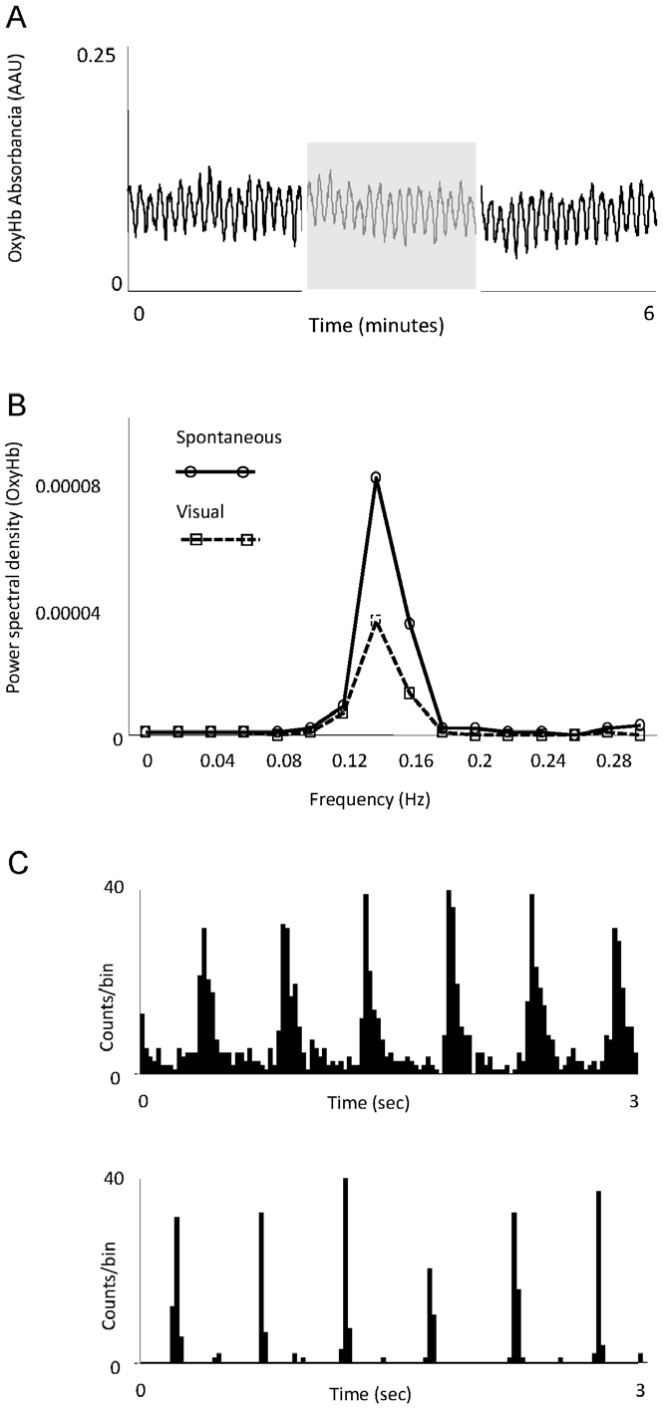
Effect of visual stimulation on oscillatory Oxyhb recordings with chloralose. A) Raw recordings of OxyHb signal during spontaneous and visual (gray bar) stimulation. B) Power spectrum analysis of the recordings shown in A. C) Peri-event raster showing the visual response of a dLGN neuron during isoflurane (top) and chloralose (bottom) anaesthesia to a drifting grating.

During chloralose anesthesia there was a total uncoupling between neuronal responses and blood flow in those periods of maximum anesthetic effect (figure 5). Visual stimulation (marked by shaded area in fig. 5A) did not evoke any increase in the recorded OxyHb signal, (the average change was 1.6% compared to 29% during non oscillatory periods under normal isoflurane anaesthesia) but a decrease in the amplitude of the oscillations, observable in the power spectrum density graph (figure 5B). During these periods, when there was no apparent augmentation in OxyHb supply, cell firing continued to be significantly modulated by sensory stimulation, even when the response of individual neurons is reduced by 60% (n = 5) in comparison to the responses of the same cells during isoflurane anaesthesia. An example of the response from one of these cells in both conditions is shown in Fig. 5C.

**Figure 6:**
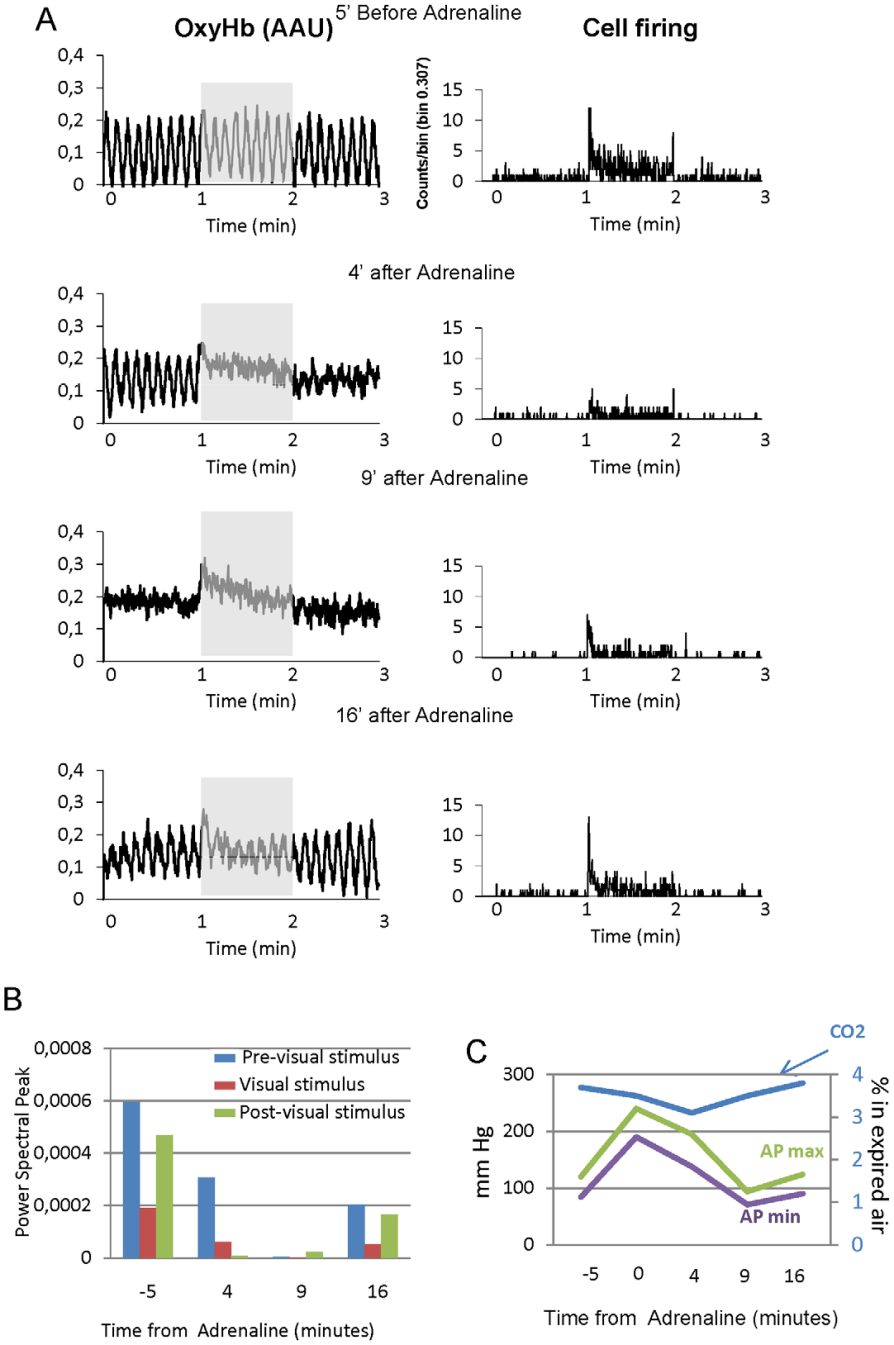
Vasomotion disruption by adrenaline. A) OxyHb recordings (left column) and firing rate of an dLGN neuron (right column) recorded simultaneously, at different time intervals following adrenaline injection. All the recordings include 1 minute of spontaneous activity, 1 minute of sensory stimulation (shaded area in the left column) and 1 last minute of, again, baseline. B) PSD peak for the recordings shown in A). The bars correspond to the different parts of the recording: pre-stimulus, stimulus and post-stimulus periods. C) Systolic and diastolic AP and expired CO_2_ values during the recordings shown in A.

**Figure 7:**
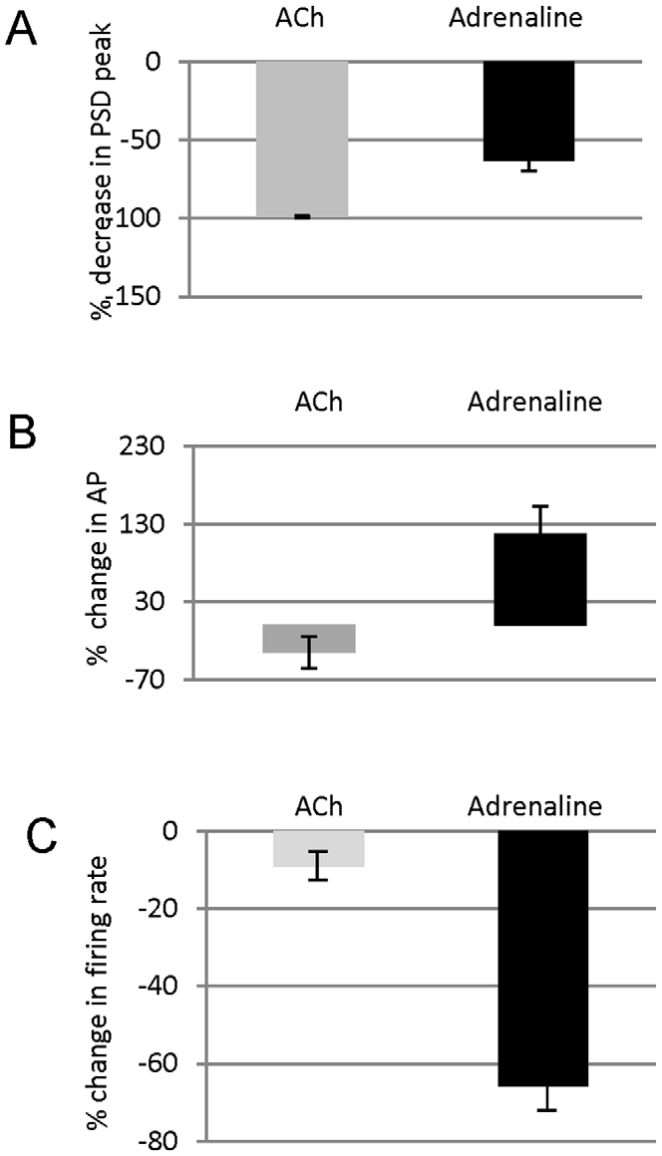
Comparison of the averaged effects of ACh and adrenaline administration. On oscillatory OxyHb signal (top), AP (center) and stimulus induced neuronal firing rate (lower).

We tested the effect of substances known to affect arterial pressure (AP) directly. Adrenaline is a well know vasopressor and intravenous administration of 0.1 mg/kg of adrenaline had a dramatic effect on oscillatory activity, reducing oscillation by 63% (n = 7, 2 cats, figures 6A, B and 7A). The maximum effect on OxyHb signal was achieved between 5 and 12 minutes after injection, during which sensory stimulation evoked the characteristic increase in OxyHb. The increase in AP was faster but shorter than the effect on oscillations: in figure 6 it is evident that 9 minutes after injection the AP (fig. 6C) is back to normal while OxyHb did not show any sign of recovering oscillation levels (figure 6B). Adrenaline produced a simultaneous decrease (average 65% ± 8 n = 7) in neuronal response to visual stimulation, temporally similar to the effect observed on OxyHb. This different behaviour of vascular and neuronal signal results in a situation of abnormal neurovascular coupling: increase in the level of oxyhemoglobin simultaneous to a decrease in neuronal response.

Acetylcholine administration (0.3 mg/kg), unlike adrenaline, decreased AP by 35% on average (fig. 7B). However, surprisingly, the effect on OxyHb signal was also a reduction in oscillations by 97% (fig. 7A, range 91 to 99% n = 5, 2 cats), in this case initially accompanied of a decrease in the intensity of the recorded signal. During this process, ACh did not change visually driven cell firing (figure 7C). Thus, pharmacological elevation or depression of AP both resulted in decreased power of the vasomotion signal, but this was accompanied by significantly different changes in the visually induced neuronal firing rate.

Finally, we could find no relationship between oscillatory activity in the OxyHb signal and other physiological parameters such as CO_2_ (normal range 3.5–4.5%).

## Discussion

Our results can be split into three components: firstly, we have demonstrated the presence of a spontaneous vascular phenomenon occurring in the visual thalamus of the cat, compatible with vasomotion. Secondly, we have shown that this phenomenon can be both induced and reduced by pharmacological manipulation. Finally, and most importantly, we have shown that during vasomotion the accepted linear relationship between blood flow and neural activity is altered, a result which must be considered when interpreting the results obtained from techniques reliant upon this relationship, such as neuroimaging.

Using a spectrophotometric signal, we have shown the presence of vasomotion-like oscillation in the microvasculature of the thalamus. This oscillatory signal shares several characteristics with vasomotion described in other species or experimental preparations: 1) the appearance under isoflurane anesthesia (52% of measured epochs) is within the mean range observed previously in anesthetized preparations [4], [23]. 2) The frequency of the oscillation, 0.14 Hz, was also in the range observed in brain circulation (0.1–0.18 Hz, [3], [24]) and oscillations lasted for periods from minutes to hours. 3) Sensory stimulation during periods of vasomotion could interfere with the oscillations (even abolishing them) but was unable to increase the Oxyhb signal to the levels seen during non-oscillatory periods. Indeed, in some circumstances, sensory stimulation simply failed to modify the oscillation. However, there was no obvious relationship between the presence of vasomotion and the other physiological parameters measured during the experiment. Oscillatory activity did not seem to be a localized event, since during periods of oscillation (lasting for several hours) we did not find regions of non-oscillatory activity at different depths in the dLGN in the same electrode track, and even in different penetrations up to 1mm apart, (although it remains possible that a local mechanism could be more important in the awake animal). The dominant frequencies observed in our preparation, around 0.14 Hz, are compatible with those of small diameter, order 3 vessels in hamster skeletal muscle [25], but our signal is likely to come from not one but probably hundreds of small microcapillaries in the area covered by our recording electrode. Hence, while it is possible that our data results from the intrinsic properties of these small vessels, we cannot discount the possibility of an input from larger vessels. The physiological role of vasomotion is unclear – it has been described as a local phenomenon [2] with potential roles ranging from a mere consequence of smooth muscle contraction (ie an epiphenomenon of some other process with no identifiable physiological role,) to a mechanism of increasing blood supply with low energy cost [7]–9. Even though vasomotion is a broadly distributed phenomenon its roles and their mechanisms may be different in different systems. Controversy even exists about how vasomotion is initiated, one example of which is nitric oxide (NO) which has been shown both to induce [12], [26]–[27], yet also disrupt, vasomotion [11], [28]–[29].

Chloralose administration induced vasomotion in a reliable and reproducible way. While the strength of the oscillation was positively related to the level of anesthesia induced by the chloralose, it is an effect that seems to be related to the anesthetic agent itself rather than the depth of anesthesia per se, since increasing the depth of anesthesia using an alternative drug, isoflurane (up to 3.5%, above full surgical anesthetic levels) did not induce oscillations. Our results with isoflurane are similar to previous studies showing an inverse relationship between level of anesthesia and vasomotion [30]–[31]. While the main reported route of action of chloralose is via activation of GABAa receptors, this is similar to many other anesthetic compounds, including isoflurane. The oscillation induced by chloralose closely followed the extent of the chloralose action, suggesting that it is a direct result of the compound, rather than a “knock-on” effect which might follow from perturbation in a physiological cascade.

We have shown that ACh and adrenaline have opposite effects on AP, but have similar, suppressive effects on vasomotion. During these effects adrenaline decreased neural responses, while ACh left them unchanged. However, peripherally measured arterial pressure may not be a good indicator of cerebral pressure, as cerebral autoregulation will also play a role which may mitigate or reverse the effects seen peripherally. ACh is normally an excitant or positive modulator of neural activity, but given intravenously it will not cross the blood-brain barrier, so that any effects observed are likely to be the result of an initial peripheral action. While previous results have shown that adrenergic stimulation induces oscillatory activity and inhibition reduces it, or prevents the induction of vasomotion by hypoxia [32], this was in peripheral circulation in muscle. The effect of adrenaline we have shown considerably outlasted the obvious change in blood-pressure, perhaps the result of secondary activation of a central response to the elevated blood pressure, with the decrease in neural activity closely following the decrease in vasomotion, rather than the change in blood pressure. During the drug administration experiments we had no direct measure of CSF pressure, but there was no obvious cerebral edema or herniation at the site of the craniotomy.

The key issue was that in response to perturbation of blood pressure, the vasomotion was decreased and the effects on cell firing different, supporting the view that there is no simple relationship between vasomotion and neuronal activity.

We have previously shown that visual stimulation evokes an increase in blood flow with a simultaneous increase in neuronal firing in the cat dLGN [33] and showed a near linear relationship between the contrast of the visual stimulus, the spiking response magnitude and blood flow. However, we have now shown that in a number of cases, the OxyHb signal can oscillate. Oscillatory movements are often evident in optical imaging or fMRI and have multiple origins; for example, respiratory and heart movements are significant but can be compensated for or removed. Vasomotion has also been shown to be able to produce low frequency fluctuations in the MRI signal [34]–[35]. However, unlike respiratory or cardiac oscillations, we have shown that the oscillation during vasomotion indicates a shift in the relationship between blood flow and neural activity such that changes in oxyhemoglobin levels do not readily predict spike firing rates, indicating a non-linear relationship and uncoupling of neural and vascular activity. Coupling between blood flow and neuronal metabolic activity is commonly accepted as part of normal brain function and it is the basis of several optical “brain mapping” techniques [36]; (see reference [37] for and excellent recent review on cerebral blood flow response). The most important optical technique is probably fMRI, which can be used to measure a blood-oxygen-level dependent signal and classically assumes a linear relationship between neuronal and metabolic responses [38], However these assumptions are being challenged [20], [38]–[43]. The mechanism(s) that regulate such coupling and the precise relationship between the different components is not well understood. We can say that the BOLD signal arises from the interplay between blood flow, blood volume and oxygenation level. Thus a local increase in oxygenated blood and a decrease (“washing”) of reduced Hb would produce the positive BOLD response, and regional decrease in blood volume and associated elevation of reduced hemoglobin would be responsible of the negative BOLD [44]. Our data will therefore have significant consequences for interpretation of results from imaging techniques based on the principle of the close coupling between neuronal and metabolic signals [20]–[21].

In sensory systems it is possible to use sensory stimulation to precisely activate or suppress neural activity, dependent upon the stimulation parameters, such as, for example, intensity, location or size. Interestingly, the position of the stimulus relative to the receptive field appears to influence oxygen responses in sensory systems [45] indicating a complex pattern of possible oxygen “responses” dependent upon both location and spatial extent of the visual stimulus, with both positive and negative responses seen (“negative” indicating decreased local oxygen signal resulting from very locally increased neural activity, which was mainly seen using very small stimuli). Further, negative bold signals have also been detected as a consequence of sensory stimulation in somatosensory cortex, taken in this case to indicate a direct vascular response to decreased neural activity [44]. We have used full field stimulation in order to induce a more “global” alteration in oxygen signal, since oscillatory activity did not seems to be a local phenomena and our stimuli are likely to have induced only excitation (albeit perhaps sub-maximally) in the majority of cells affected. However, studying the effects of spatially limited stimuli or stimuli with different properties (i.e. temporal and spatial frequencies different to those which evoked the oscillatory response) is interesting idea for future studies.

Local alterations in neurovascular coupling have been demonstrated in several different models. For instance, Leithner et al [46] showed, in the rat somatosensory cortex, a pharmacologically induced reduction in the cerebral blood flow response to functional activation by approximately two-thirds, while neuronal activity and cerebral metabolic rate for oxygen remain largely unaffected. However these manipulations did not measure the relationship between blood flow and neural activity across a dynamic range (ie is the relationship linear or non-linear), merely indicating that the response induced by changes in neural activity probably includes a substantial “safety margin” [46], which may be equally present in all responses to sensory activation. Pathological change, either a result of disease or manipulation (as above) has the potential to alter neurovascular coupling significantly (see Lindauer et al. 2010 [47] for a review). Notably, we show here changes in neurovascular coupling as a consequence of vasomotion, considered to be a physiological, rather than pathological, phenomenon.

Finally, Niessing et al [48] found spontaneous fluctuations in the intensity of the spontaneous signal correlated with the frequencies of recorded local field potentials (LFP) and poorly with action potentials. We recorded individual neurons and it would be interesting to study LFP in our model or to simultaneously record several neurons in order to study synchrony during the appearance of vasomotion.

## Materials and Methods

### Animal preparation

#### Ethics Statement

All the procedures followed the guidelines of the Spanish Physiology Society and the International Council for Laboratory Animal Science and the European Union (statute nr 86/809). Procedures were approved by the ethics committee of the University of Coruña (CE-UDC30/1/09).

17 adult cats of either sex were prepared following standard procedures used in our laboratory [49]. Animals were food-deprived before anaesthesia. Anaesthesia comprised isoflurane (1.5–2% for surgery, 0.2–1% for maintenance) in nitrous oxide (70%) and oxygen (30%). The trachea was cannulated, an intravenous (i.v.) line inserted, and a craniotomy performed for thalamic recording. Animals were held in a stereotaxic frame and paralyzed with gallamine triethiodide (loading dose of 40 mg, maintenance 10mg/kg/h i.v.) to prevent eye movements. End-tidal CO_2_ levels, electrocardiogram (ECG), AP, and electroencephalogram (EEG) were monitored continuously throughout the experiment. The rate and depth of artificial respiration was adjusted to maintain end-tidal CO_2_ levels at 3.5–4.5%. The level of isoflurane was chosen to achieve a state of light anaesthesia typically used in visual experiments. Once a stable state was achieved, any variations of the monitored parameters commensurate with a change in the depth of anaesthesia were compensated by alterations in the level of isoflurane. Wound margins were treated with lidocaine hydrochloride administered subcutaneously. In 2 animals the anaesthesia was switched during the experiment. These animals were initially anaesthetized with Isoflurane, and after obtaining a robust non-oscillatory signal, Isoflurane was removed and a bolus of alfa-chloralose (60 mg/kg, i.v. dissolved by heating in physiological saline) was injected. A new bolus was administered when physiological parameters indicated it as necessary, typically each 6-8 hours.

Ears bars of the stereotaxic frame were coated with lidocaine gel. The eyes were treated with atropine methonitrate and phenylephrine hydrochloride, protected with zero-power contact lenses, and brought to focus on a semiopaque tangent screen 57cm distant using appropriate, trial-case lenses. Visual stimuli were viewed monocularly through 3-mm artificial pupils. At the end of the experiment the animal was killed by anaesthetic overdose.

### Electrophysiological and spectroscopy recordings

Extracellular single units and multiunit activity (MUA) were recorded (Plexon Inc, Dallas, TX) using tungsten microelectrodes, diameter 150 µm, impedance 10 MΩ (FHC Bowdenheim USA). Spectroscopic measurements of OxyHb were obtained through two optical fibers attached to the electrode which allowed us to record both single unit activity and blood flow from the same location [33]. Fibers model FCB-UV100-3.0-2SMA, diameter 100 µm. The electrode was attached to the fibers under microscope with dental acrylic. Distance from the electrode tip to the fibers end was between 30 and 50 µm. The electrode tip must be above the fibers in order to avoid shadows that could interfere with the spectroscopic analysis.

Light in the range of 460–800 nm from a halogen lamp was passed through one optical fiber and scattered light collected by the second. Output was directed to a linear CCD detector device (Oceans optics, Eerbeek, Netherlands) via a compact built-in monochromator. The OxyHb levels, expressed in absorbance arbitrary units, were calculated as follows:

(((Absorbance_576nm_)-0.55*(Absorbance_576nm_)-0.45*(Absorbance_576nm_))/15448)*150.

All observations were made in the dLGN A laminae at a visual location less than 12° from the area centralis. The sample of single units included X and Y cells that were differentiated on the basis of a battery of standard tests, including the null test (linearity of spatial summation), receptive field size and eccentricity, type of response to flashing spots, and presence or absence of shift effect 50–52. Waveforms and time stamps were stored (Plexon Inc, Dallas, TX) and off-line sorting was used to assess adequate isolation of spikes. This allowed us to isolate individual waveforms from noise with certainty. Spectroscopic observations were always coupled with single unit recording. Experiments lasted up to 2 days and during many of the periods when no oscillations were present extracellular recordings were used for other protocols, unrelated to vasomotion.

### Visual stimulation

Computer-controlled visual stimuli (Lohmann Research Equipment, Germany) were presented monocularly on a monitor (mean luminance of 14 cd/m^2^, contrast 0.6, refresh rate 128 Hz) 57 cm from the eye [49]. Visual stimuli consisted of full field sinusoidal drifting gratings centred on the receptive field of each cell. The spatiotemporal properties of the stimuli were optimally set for each cell.

### Experimental design

Experiment started with the correct location of the spectroscopy probe in the dLGN assessed by visual responses obtained from the attached electrode. Both elements were slowly displaced until a cell was isolated. Neuronal response was characterized in terms of receptive field, and preferred spatial and temporal frequencies. At this point we simultaneously established a base line for cell spontaneous activity and spectroscopy signal and studied the effect of visual stimulation on both recordings. A common stimulus protocol was 1 minute of spontaneous activity, 1 minute of full field drifting grating followed by another minute of spontaneous activity. This protocol allowed us to set stables floor values and study the different components of the responses even in the presence of strong oscillatory activity. This is considered through the paper as one recording. The recording could be repeated at different time intervals (in order to confirm the presence or not of oscillatory signals) or during several pharmacological manipulations (in order to try to study the mechanisms behind the oscillations). If at some point the neuronal signal was lost, the probe, plus the electrode, was displaced until finding a new cell. Displacements of the probe were also made during oscillatory periods to study the spatial extension of the oscillation. Occasionally we recorded MUA in order to study the relationship between metabolic signal and population responses. Several penetrations were made in each experiment, with several recording in each of them.

### Pharmacology

The effects of i.v. administration of Acetylcholine (ACh 0.3 mg/kg) or Adrenaline (0.1 mg/kg) on oscillations were measured. Drugs were purchased from Tocris (United Kingdom) or SIGMA (Spain). Both drugs were dissolved in 0.3 ml of sterile saline and infused as a single bolus.

### Data analysis

Data in text and figures are presented as raw values or percentage of the baseline value unless otherwise indicated. OxyHb values were averaged during periods up to two minutes of spontaneous and visual evoked activity. Statistical analyses of the effects of various experimental interventions were performed using a Student t-test. P values<0.05 were considered statistically significant. Autocorrelograms of neuronal activity and coherence analysis of both neuronal response and OxyHb signal, as well as power spectrum density (PSD) analysis was made with commercial software (NeuroExplorer).

## Supporting Information

Figure S1 **Effect of increasing isoflurane on oxyHb and neuronal evoked signals.** Average values from 4 simultaneous cells/recordings ±SEM. Both signals showed a linear decay directly related to the increase in level of the anesthetic, with R^2^ values of 0.9318 and 0.9133 respectively.
(TIF)
