# Supplementary figures and images for: Vasomotion and Neurovascular Coupling in the Visual Thalamus In Vivo
Source: PLoS One. 2011 Dec 9;6(12):e28746. doi: 10.1371/journal.pone.0028746 (PMC3235153; doi:10.1371/journal.pone.0028746)

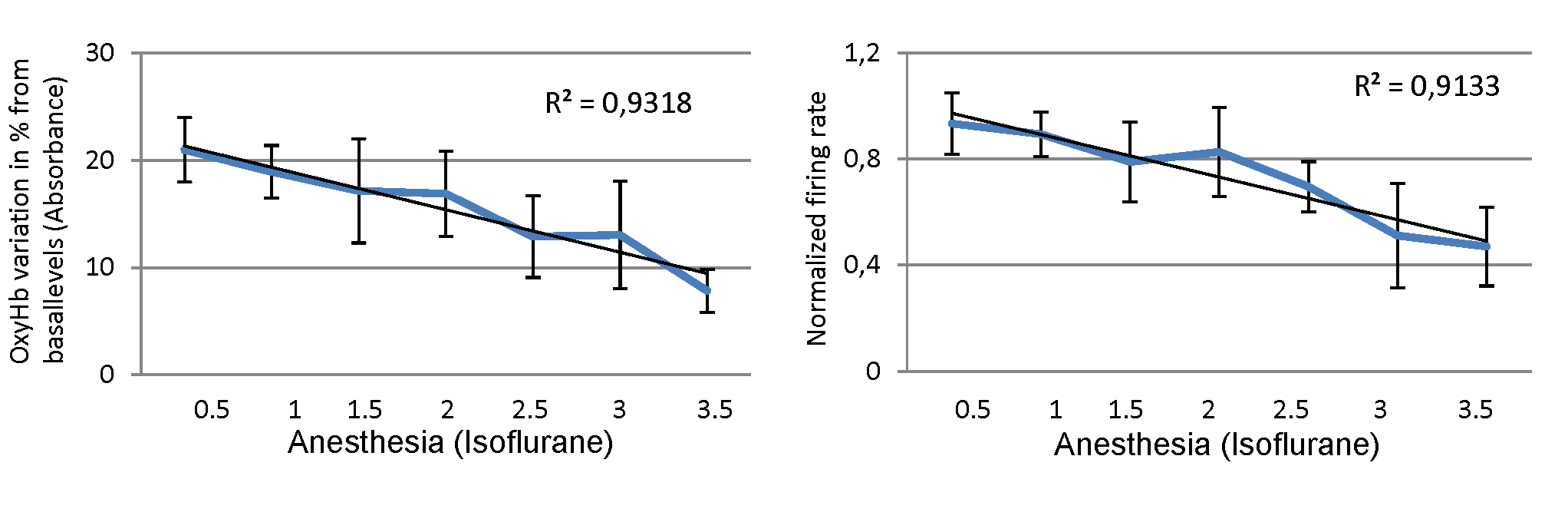

Supplement: Figure S1 — Effect of increasing isoflurane on oxyHb and neuronal evoked signals. Average values from 4 simultaneous cells/recordings ±SEM. Both signals showed a linear decay directly related to the increase in level of the anesthetic, with R2 values of 0.9318 and 0.9133 respectively. (TIF) [file pone.0028746.s001.tif]
